# Monitoring of Thermal Comfort and Air Quality for Sustainable Energy Management inside Hospitals Based on Online Analytical Processing and the Internet of Things

**DOI:** 10.3390/ijerph191912207

**Published:** 2022-09-26

**Authors:** Hugo O. Garcés, Claudia Durán, Eduardo Espinosa, Alejandro Jerez, Fredi Palominos, Marcela Hinojosa, Raúl Carrasco

**Affiliations:** 1 Departamento Ingeniería Informática, Universidad Católica de la Santísima Concepción, Concepción 4090541, Chile; 2 Departamento de Ingeniería Industrial, Facultad Ingeniería, Universidad Tecnológica Metropolitana, Santiago 7800002, Chile; 3 Department of Electrical Engineering, Faculty of Engineering, Universidad Católica de la Santísima Concepción, Concepción 4090541, Chile; 4 Saval Pharmaceutical, Santiago 8640002, Chile; 5 Departamento de Matemática y Ciencias de la Computación, Universidad de Santiago de Chile, Santiago 9170022, Chile; 6 Departamento de Tecnología Médica, Facultad de Medicina, Universidad de Concepción, Concepción 4070409, Chile; 7 Facultad de Ingeniería y Negocios, Universidad de Las Américas, Santiago 7500975, Chile

**Keywords:** air quality, internet of things, hospitals, thermal comfort

## Abstract

There is a need to ensure comfortable conditions for hospital staff and patients from the point of view of thermal comfort and air quality so that they do not affect their performance. We consider the need for hospital employees and patients to enjoy conditions of greater well-being during their stay. This is understood as a comfortable thermal sensation and adequate air quality, depending on the task they are performing. The contribution of this article is the formulation of the fundamentals of a system and platform for monitoring thermal comfort and Indoor Air Quality (IAQ) in hospitals, based on an Internet of Things platform composed of a low-cost sensor node network that is capable of measuring critical variables such as humidity, temperature, and Carbon Dioxide (CO_2_). As part of the platform, a multidimensional data model with an On-Line Analytical Processing (OLAP) approach is presented that offers query flexibility, data volume reduction, as well as a significant reduction in query response times. The experimental results confirm the suitability of the platform’s data model, which facilitates operational and strategic decision making in complex hospitals.

## 1. Introduction

The United Nations has proposed the need to focus the efforts of states, companies, and communities on the Sustainable Development Goals (SDGs). These global goals aim to eliminate poverty, protect the planet, and ensure prosperity for all. In order to comply with the SDGs, actions and policies must be generated to improve the health of the population, reduce the gender gap, access more affordable and cleaner energy sources, and improve the quality of work and environmental conditions [1]. In countries with lower income, the achievement of the SDGs is more difficult to meet, because there is a direct relationship between Gross Domestic Production (GDP) per capita and the country’s spending on energy, as the GDP decreases there is a per capita increase in energy poverty, which can be measured by multiple indicators [2]. However, it can be verified that a lower GDP is directly related to energy poverty, which means that there is less capacity of households to meet basic needs related to the use of energy resources [3]. It is important to measure social policy and the vulnerability of certain groups through an energy justice perspective [4]. Despite the efforts that promote renewable and clean energy policies, there is a lack of institutions that make effective investment decisions that reduce the negative externalities generated by the large amount of air pollution, Greenhouse Gas (GHG) emissions, and other factors [5].

It is observed that the sectors that consume the most energy are commerce, public and private organizations, as well as the residential sector, due to the operation of Heating, Ventilation and Air Conditioning (HVAC) systems in buildings and real estate. The use of HVAC systems is intended to achieve a comfortable indoor environment and high air quality for the occupants [6,7,8]. Therefore, the demand for energy from HVAC systems shows a growing trend and, at the same time, practices are required that improve its use together with clean generation and conversion methods that guarantee sustainability [9,10,11].

It is necessary that the authorities generate sustainable policies that meet the energy needs of the population and meet the purposes of reducing energy poverty worldwide and reducing GHG emissions that affect human activities and the environment [3,12,13].

In response to the growing demand of building occupants who need an interior environment whose thermal comfort and air quality are suitable for their activities, it is necessary to improve the operation or installation of air conditioning systems with a better design of the envelopes and the isolation [14,15,16]. The quality of the indoor environment is important as it affects health, cognitive performance, and productivity [17,18]. The results of different studies show that thermal comfort is rated as the most relevant aspect compared to air quality, visual, and/or auditory comfort [19,20]. Due to the foregoing, the development of methods and technology is required to contribute to obtaining a comfortable environment for the occupants of the property, balancing thermal comfort, Indoor Air Quality (IAQ), and energy consumption; but without omitting externalities produced by the use of these energy-consuming systems, such as the carbon footprint and the economic viability of the operation of these intelligent building monitoring systems [21,22], accounting for a system of multi-objective energy optimization [23]. Together with the development of technologies that are framed in the monitoring of critical infrastructure [24], measures must be promoted that account for a policy that favors equity in terms of access to energy [25].

### Thermal Comfort and Air Quality in Hospitals

Currently, the air conditioning of hospitals is a process that consumes a large amount of electrical and thermal energy. In air conditioning, only the variables of temperature and humidity are considered, which fits with the definition of thermal comfort contemplated by the ISO 7730 standard [26]. The ASHRAE 55-2013 standard [27] defiNES thermal comfort as the perception that represents the thermal condition of a satisfactory environment. Both definitions are quite broad, as each person may have a different perception of thermal compliance for the same environment. In general, thermal flexibility is a condition of neutrality which means that the person does not feel too cold and not too hot. The latter represents an energy balance between the body and the environment, which implies that the body can balance the heat gained with the heat removed.

On the other hand, the non-neutral condition is corrected by HVAC systems. This verifies the close relationship between energy consumption in a building and thermal comfort. If the latter is optimized and neutrality is achieved with lower energy consumption, energy efficiency [28,29,30] is achieved. The work developed by Fanger [31] proposes the calculation of thermal comfort considering the following indoor variables: (i) level of physical activity, (ii) insulation level of the clothing, (iii) mean radiant temperature, (iv) Relative Humidity (RH), (v) temperature, and (vi) air velocity. From these measurements, two indices are calculated: Predicted Mean Vote (PMV), which estimates the average thermal sensation, and the Percentage of People Dissatisfied (PPD).

Hospital facilities are one of the types of buildings where it is more difficult to control thermal comfort and IAQ due to (i) different HVAC and air conditioning technologies, (ii) changing internal infrastructure, depending on requirements, (iii) heterogeneity of its occupants (according to their clothing characteristics, physical activity, etc.), and (iv) HVAC and air conditioning systems depend on two types of energy: electrical and thermal [32,33]. In these facilities, it is necessary to develop solutions that allow adequate thermal comfort in the rooms of hospitalized and outpatient patients (adults, children, and medical personnel), waiting rooms, procedure rooms, and laboratories [32]. In particular, their specific requirements should be considered, especially if they are burn patients or patients with an impaired immune system due to disease or medication. It should be noted that to control temperature, humidity, and ventilation and reduce the proliferation of both bacteria and viruses in the facilities, the specifications of the ASHRAE standard are used [34].

On the other hand, it is necessary to develop intelligent ventilation systems that make efficient use of electrical energy and improve IAQ [20,35]. Such is the impact that, according to the World Health Organization, around 99,000 deaths in Europe and 81,000 in the United States are attributed to inadequate IAQ in residential buildings (Carbon Dioxide (CO_2_) > 500 ppm) [35,36].

In China and the Netherlands, environmental variables in hospitals were measured and analyzed, concluding that the HVAC system needs to achieve different environmental conditions in each of the rooms because they have to meet humidity requirements, specific temperature, and IAQ [37,38]. It is worth mentioning that in smart cities, public and private actors work collaboratively to monitor air quality using monitoring systems with microcontrollers that connect sensors that have, among others, the input parameters temperature, RH, Carbon Monoxide (CO), and Ethanol (C_2_H_5_OH), Volatile Organic Compound (VOC) [39,40]. It is important to exercise effective control since a high concentration of pollutants CO_2_, Sulfur Dioxide (SO_2_), Nitrogen Dioxide (NO_2_), Nitrogen Oxides (NO_x_), Particulate Matter 10 micrometers or less in diameter (PM_10_), and Particulate Matter 2.5 micrometers or less in diameter (PM_2.5_) can cause short- and long-term diseases [41,42].

Thermal comfort, energy efficiency, and air quality in hospitals are issues that are closely related. For an organization, it is important to plan the operation of HVAC systems in the short term and make long-term decisions regarding automatic control systems or expert operators, in order to obtain direct benefits by reducing energy consumption (thermal and electrical) and indirect, by improving working conditions such as the risk of spreading diseases in patients and workers, as well as in the planning of energy investments (coatings and thermal insulation, installation or repair of HVAC systems) [32,43,44,45].

With technological development, instrumentation and automatic control systems appeared [24]. For the monitoring and control of thermal comfort and air quality in hospitals, platforms based on the Internet of Things (IoT) [46,47] are currently being used, which are characterized by (i) having a hierarchical structure where each layer has independent functions, such that the interaction between layers is done through signals processed throughout the platform, until the information to support the final user’s decision making is displayed in the operation interface; (ii) the intelligent sensors that make up the perception or sensing layer measure the variables of interest and are built with components from multiple technologies together with an embedded computing system that allows the platform’s intelligence and computing capacity to be distributed; (iii) managing service quality requirements, maintaining a limited level of energy consumption and data transmission; (iv) a ubiquitous communications system that uses the sensing layer and is able to combine multiple forms of digital communications (wired or wireless Ethernet, Global System for Mobile, among others) where the data travels in the dedicated or proprietary network; (v) it has stable interfaces between applications, which allows the independent interaction of the hardware and the operating system of each computing device; (vi) it supports the presence of digital twins to predict or estimate the phenomenon of interest in the face of different changes in its environment.

However, it requires paying attention in design and operation to issues related to privacy and data processing.

## 2. Chilean Public Hospital System

### 2.1. Classification of Public Hospitals

Chile, being a geographically elongated country and centralized in its administration, the distribution of hospitals is complex. It is difficult to satisfy the needs of the inhabitants and build hospitals in each locality [48]. Public health establishments serve approximately 80% of the population and are managed by the Ministry of Health [49,50]. A hospital is understood as an enclosure and its infrastructure that is intended to provide health benefits for the recovery, rehabilitation, and palliative care of sick people, as well as collaborate in promotion and protection activities, through outpatient actions or in closed care [51].

According to Decree Law 140, hospitals are classified as high, medium, or low complexity, depending on their function within the healthcare network, diagnostic and therapeutic support services, and the degree of specialization of human resources [51]. During the year 2021, it was observed that Chile had 63 public hospitals of high complexity, 32 of medium complexity, and 98 of low complexity [52].

Currently, hospitals are flexible structures, with healthcare network characteristics, depending on the type of activities, levels of complexity, and specialties assigned by the network manager [53]. There are macro-networks at the regional and national levels in which patients can be referred according to their level of complexity. The most complex oNES are distinguished by being self-administered managers, they work in a network, they are decentralized, they are autonomous in their decisions, and they cover their entire population [52].

Currently, hospitals are governed by the Chilean energy policy projected until the year 2050, which aims for all new buildings to have Organisation for Economic Co-operation and Development (OECD) standards for efficient construction, with intelligent energy control and management systems, which reduce greenhouse gas emissions [54]. It should be said that despite the public efforts that have been made in this area, a more detailed characterization of thermal comfort in the Chilean hospital sector remains to be developed. This was observed in the audits carried out by the Energy Sustainability Agency during the years 2014–2016, due to the fact that the surveys conducted to users did not focus on key issues of thermal comfort such as temperature, humidity, indoor wind speed, and outdoor climate [55].

### 2.2. Care Protocol of a Chilean Public Hospital of High Complexity

Public hospitals serve people, also called users, who choose the health service closest to their location [56] and Law No. 20,584 regulates the rights and duties of users, professionals, and workers who participate in the care process [57].

**Figure 1:**
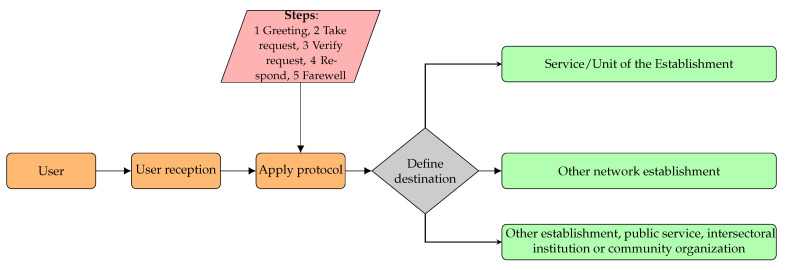
Protocols used in the process of attention.

As shown in Figure 1, the services provide different types of care. In particular, people with disabilities (according to their physical, psychosocial, cognitive, visual, or hearing condition) or migrants have special treatment [56,58]. Depending on the requirements of the users, they could be referred to specialized health establishments.

### 2.3. Characterization of the Energy Matrix in a Highly Complex Hospital

The determination of the long-term energy matrix of a hospital is a complex process that is based on the analysis of climatic conditions and the availability of energy sources [59]. For the above, it is necessary to evaluate the relevance of the different types of supplies: (i) oil, (ii) gas, (iii) pellets, (iv) biomass or other, (v) non-conventional renewable energies, (vi) electricity, and (vii) other energy sources available on site. Each type of supply needs enclosures or spaces for its storage, disposal, or distribution, which is dimensioned according to the geographical area, the useful life, the rates, the operational costs, and the environmental pollution plans [60,61].

In high-complexity hospitals, the most used energy source is electricity, which comes mostly from the National Electricity System (NES), which has a coverage of 98.5% of the national territory [62]. Various systems that consume energy are integrated into the enclosures: (i) lighting, (ii) heating, (iii) information technology, (iv) cooling, and (v) auxiliary supplies, with equipment associated with electric motors, pumps, etc.

On the other hand, it is possible to control the management of energy consumption in a highly complex hospital, with indicators that measure energy consumption with respect to an occupied bed, a square meter, and air conditioning, among others [63].

### 2.4. Motivation

The motivation for this work arises from the strategic need to implement Information and Communications Systems (ICS), which store, process, and transfer environmental data in a highly complex public hospital in the Bío-Bío region. With the development of ICS, it is possible to obtain benefits in the management of the facilities and improve the attention of users by reducing bureaucracy, as well as optimizing the allocation and use of economic and energy resources and increasing human capital.

With the implementation of information systems, it is possible to make effective and efficient decisions according to the specific energy needs related to the large amount of data that is handled on the thermal comfort and air quality of the hospital premises. Likewise, it is possible to comply with the requirements of the “Regional Development Strategy” and the “Energy Policy 2050”, which seek to generate technical instances for the management of sustainable and intelligent energy resources [64,65].

**Figure 2:**
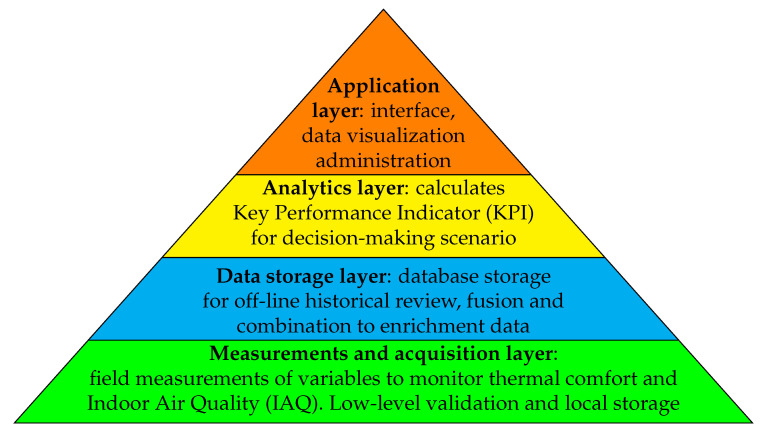
IoT platform as a hierarchical structure.

To facilitate decision making, it is necessary to generate new information and knowledge that helps the administration of hospital facilities. For this, as shown in Figure 2, it is required to create an IoT platform that has a hierarchical structure where a large amount of data are collected, processed, analyzed, measured, and displayed. At the base of the pyramid are the smart sensors, which provide information on the energy and IAQ required to carry out the different hospital processes.

On the other hand, to control the hospital energy process and generate greater personal well-being and energy savings, it is necessary to create a real-time monitoring system that, through the KPI, compares whether the perception of satisfaction or comfort of the users is in accordance with the thermal condition of the environment and the quality of the indoor air. Another reason why it is important to implement indicator-based monitoring systems is that the hospital campuses must implement the Sustainable Building Certification (SBC) hospital version [66], which requires buildings to achieve adequate levels of environmental quality interior and thermal comfort, in such a way that the use of resources is optimized in operations and the generation of both waste and emissions is reduced.

It should be said that the first months of the COVID-19 pandemic in 2020 revealed the disadvantage of not having monitoring systems. Indeed, the air conditioning and quality systems, because they only enter or recirculate air, became obsolete due to the lack of the ability to ensure that the indoor air is of good quality $\left(<350\;\mathrm{ppm}\;{\mathrm{CO}}_{2}\right)$ [67]. This is how the installation of a system of sensor nodes [68] that allows the measurement of air quality and thermal comfort in hospitals, can help the energy manager or air conditioning operator to improve not only energy performance but also to improve quality of the air inside this type of building [66].

## 3. Materials and Methods

In the monitoring of thermal comfort and indoor air quality in a hospital, the construction of a platform IoT is based on the characteristics of the processes and indicators, which can be related to variables linked to the type of communication, energy consumption, number of nodes and the location of sensor nodes, and data transmission, among others. To identify the key aspects required for the specifications, field visits must be made and the hospital’s electrical and structural plans must be reviewed.

It is important to obtain the hardware specifications related to the communication techniques and the electromagnetic compatibility of the data transmission network. In addition, it is required to know the software definitions that are linked to the operation interface, together with the users of the platform. It should be said that the software specifications define the variables to be displayed on the main screen, the number of tabs, the development of a customized report, the privileges of the user and administrator profile, and the User Interface (UI). The UI is developed according to the Geographical and Environmental Database Information System standard, which integrates ergonomics, human–machine interface design, and systems engineering to display a screen that adds value to the end user [69].

The present research works with the information of the hardware and software operational requirements, together with the definitions of basic engineering and design of the sensor node that represents the theoretical/technological support of the proposed solution.

**Figure 3:**
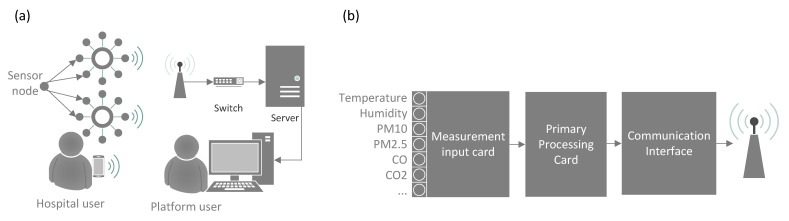
Schematics of IoT platform monitoring of thermal comfort and indoor air quality in hospitals: (**a**) Basic engineering of platform components; (**b**) basic engineering sensor node.

The results of the detailed engineering in the development of the IoT platform are used in the study, according to the model that divides the development into phases [70]: (1) Strategic definitions, (2) Concept design, (3) Detail design, (4) Verification and testing, and (5) Final disposition. Figure 3a,b represent the conceptual design that will be developed to build the IoT platform and the sensor node. The design includes parallel hardware and software development, validation of laboratory testing and field testing of data acquisition by the sensor nodes, processing of measurements, storage, and display of trend graphs on the IoT platform. The detailed design includes the development of a global energy modeling of each enclosure from the available measurements.

Then, the following objectives will be achieved:

1. Design a cyber–physical conceptual model that can be implemented in the hospital and that allows efficient decision-making.
2. Create conceptual and logical models of multidimensional databases that will allow the subsequent implementation of computer systems.

Finally, a case study will be developed in which it will be investigated whether it is possible to generate a monitoring system that measures thermal comfort and air quality inside the Dr. Guillermo Grant Benavente Regional Clinical Hospital in Concepción. It is worth mentioning that the analysis of air quality will be based on the European standard [67] and that for the study of thermal comfort the manual of recommendations on sustainable hospital building will be used, a key requirement to obtaining the Chilean Certification of Sustainable Buildings cite Institute of Construction 2017.

## 4. Hospital Cyber–Physical System

### 4.1. IoT Platform as a Cyber–Physical System

With the emergence of IoT platforms or systems, a bridge is established between the virtual and physical dimensions of system architecture. In this sense, it can be inferred that hospital care processes, together with the presence of smart sensors, form a Cyber-Physical System (CPS) from the monitoring perspective. The CPS are of wide research interest as a transdisciplinary topic, including communication technologies, computer science, electrical-electronic engineering, instrumentation and control systems, instrumentation, etc.

**Figure 4:**
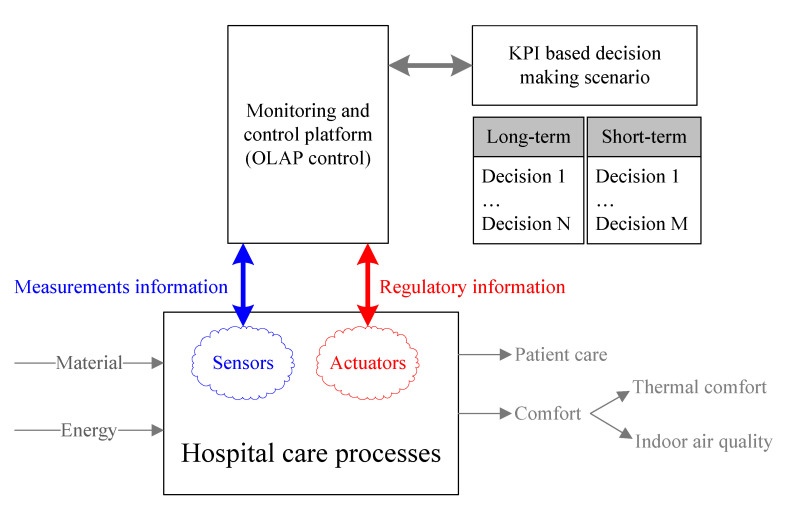
Process of hospital attention: Cyber-Physical System (CPS) On-Line Analytical Processing (OLAP); Key Performance Indicator (KPI).

From the CPS perspective, Figure 4 shows the main components of the cps, which makes it feasible to support short-term and long-term thermal comfort and IAQ decision-making scenario, considering the information available from the IoT platform and the calculation of performance indicators. The components in red are outside the scope of this study since adjustment actions are not carried out in the hospital. In addition, this data management model based on On-Line Analytical Processing (OLAP) allows for a flexible structure in terms of the KPIs to be calculated on the platform to satisfy the end user information requirements, which may vary in their formulation over time, as more measurements and data are available on the platform from the smart sensors.

### 4.2. Design of the Cyber–Physical System

**Figure 5:**
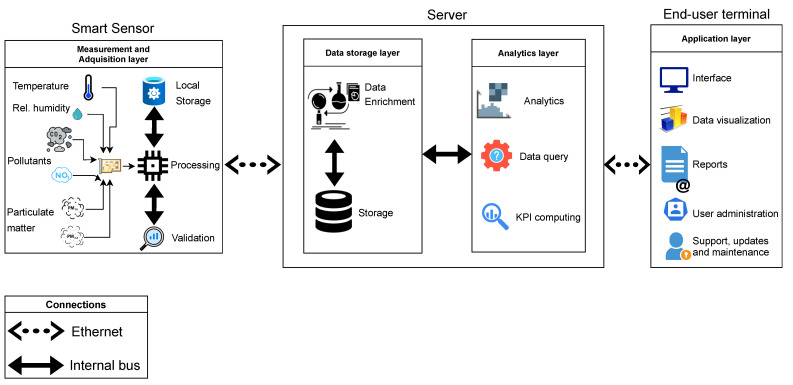
Flowchart.

As can be seen in Figure 5, the platform’s data flow begins with the acquisition of data on temperature, humidity, particulate matter, and IAQ gases, which are measured through the components that make up the smart sensor. The reading of the sensors is done through the connection with an Arduino UNO development board every 60 s and with its respective timestamp, which establishes the instant of measurement. Said data acquisition executed by the Arduino UNO development board is transmitted to a low-cost Raspberry PI card, through a direct serial connection inside the smart sensor.

The Raspberry runs a Open Platform Communications Unified Architecture (OPC UA) Server that saves the data obtained from the Arduino, verifying first that it has a timestamp and the quality of the data, which in this case is defined by two possible states (‘GOOD’ or ‘BAD’). Including communications via OPC UA allows obtaining multiple advantages, among which is the incorporation of a self-contained information system model, which is easily expandable from the levels closest to the sensors to the highest levels of organization management [71].

## 5. Monitoring System for Thermal Comfort and IAQ in a Hospital

### 5.1. Main IoT Platform Components

Internet of Things (IoT) platforms or systems are characterized by the connectivity of multiple devices that have a communication component to connect to the Internet and through it send the information they collect to a platform [39,40]. They are designed and operate as a hierarchical structure, where each layer has independent functions along with their respective functional modules and the information is transmitted through interconnection signals (typically digital) [72,73].

Research has used different types of technologies that have advantages and disadvantages in terms of accuracy, availability, difficulty of implementation, and costs in hospitals [74]. For efficient management, data sources that receive information from IoT sensors are integrated and sent to end users on a platform [75,76]. Current healthcare information systems handle large amounts of data that need to be processed with technologies and made available on platforms so that physicians can make decisions informed of the user’s condition [77,78].

**Figure 6:**
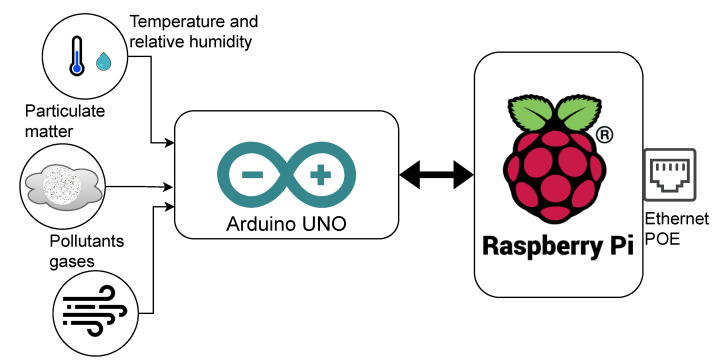
IoT smart sensor basic structure.

The smart sensors shown in Figure 6 are characterized by having a low cost per unit, performing temporary storage tasks, and validating the data that is measured, together with encapsulating and transmitting at the physical layer, via Ethernet with OPC UA, the multiple measurements of the variables of interest to execute the hospital monitoring in the platform. The data quality is assessed by computing a small validation routine which consists of verifying that absolute variation between two consecutive measurements is less than a threshold, and comparing the measurement according to the sensor range. If both tests are ok, then the measurement is correctly validated; otherwise, it is replaced by the previous measurement. Thermal comfort is computed according to the Fanger method, taking into account air temperature and humidity and the most relevant values to calculate thermal comfort. In more detail, the smart sensor considers the following features: air temperature and humidity range according to indoor conditions with the DHT22 sensor, IAQ variables are obtained with SDS-011, MICS-6814, and MG-811 low-cost sensors, and the final cost of the smart sensor is about USD 400. Designing a low-cost sensor is an important issue in order to reduce energy poverty gaps related to technology access to monitor the environmental conditions that patients, visitors, and hospital workers are exposed to. More details about the smart sensors to perform the field measurements can be found in [68].

Considering the need to carry out continuous monitoring of the set of indicators described in Section 5.3 and the capture capacity of the platform of sensors destined to collect data for the computation of indicators related to the thermal comfort of hospital facilities, a continuous sampling process of variables that help determine the state of thermal comfort and IAQ in the hospital was carried out. In particular, the following variables were sampled: temperature, humidity, PM_10_, PM_2.5_, CO, CO_2_, VOC, NO_2_, and C_2_H_5_OH. Other necessary variables to establish thermal comfort are air speed, clothing insulation, and metabolic rate. However, evidence indicates that air temperature and humidity are the most relevant variables (more details can be found in [25,79,80]).

The sampling control of the sensor network is carried out in the node, which recursively activates the script that controls the measurement and local validation of the measurements. For the local validation of the measurements obtained by the sensor node, TESTO 480 is used as a standard instrument. Then, the measurement is temporarily stored in the sensor node until the data is transmitted to the server for data enrichment by calculating PMV and PPD with the Fanger method [68]. Parametrization of clothing insulation and physical activity to calculate PMV and PPD is performed by observing during a 7-day experimental campaign, for 2 h a day, what the predominant activity in the sensor location was, including medical staff, administrative staff, patients, or visitors. This procedure allows us to approximate the metabolic activity of the occupants. To determine the average level of clothing insulation, the season of the year is considered. From the above, average values of clothing insulation and metabolic heat rate are obtained.

The enriched measurements of PMV and PPD allow estimating the average thermal comfort in the hospital, considering that the parameterization of the sensors included an experimental campaign where a different metabolic rate is entered for each sensor node. This difference in metabolic rate is a product of each sensor node being installed in locations where office work, light-motion work, or rest are performed, which leads to differences in energy expenditure and in the sensation of thermal comfort (see Table 1).

Regarding IAQ, each stored variable provides information of interest on critical variables such as the level of occupancy of the property, and level of air purity, among others.

It is worth mentioning that the information on the critical variables will be generated from indicators 4, 5, 6, 7, 8, and 9, as shown in Section 5.3. In addition, the estimated number of people using or circulating on average around the sensor node will be considered.

### 5.2. Data Processing and Indicator Management Sub-System

Considering the needs of the different actors that intervene in the control of environmental conditions in places of public use as well as in the improvement processes, it is necessary to offer and ensure the availability of a record of key indicators regarding various types of environmental variables relative to people’s sense of well-being and the state of the different factors that make up public spaces, particularly those related to air quality. Since this set of indicators is diverse in terms of the characteristics of the measurements (discrete or continuous; objectivity or subjectivity; instrumentation, measurement, and recording mechanisms; etc.), it is necessary to organize the information so that it reflects all these factors and their interrelationships, as well as having the ability to access or process this information at the appropriate times and with times according to the needs.

The implementation of an information system with multidimensional characteristics is proposed, which allows responding to the previously described needs. The system consists of a technological tool that provides indicators of environmental information available online and with direct access, which allows visualizing or obtaining historical sequences of values related to specific precincts or to groups of precincts, in closed spaces of public or private urban infrastructure, with the purpose of helping managers or researchers to achieve stable environmental comfort conditions, propose improvement plans, the rational use of air conditioning resources, evaluate initiatives related to comfort, as well as the efficient use of energy resources related to users and the workers of the enclosures.

The system includes indicators that provide information related to environmental comfort and energy consumption, in the following dimensions:

- Physical location: Refers to buildings within well-defined limits, which are made up of multiple and different types of dependencies, which are intended to provide public use services for different types of people.
- Spaces: They are specific dependencies belonging to and contained within an enclosure, which are used by certain types of people (users). Usually, each space has one or more specific functions designed to satisfy all or part of the needs of the direct or indirect users of the site.
- Activities: These are specific functions that are within the work or service, which are developed by people or machiNES under the supervision of specialized personnel and have the purpose of providing direct or indirect service for the benefit of users to whom the venue is dedicated.
- Temporality: Provides temporal anchoring information that allows all the information contained in the system to be accurately located in time, to reflect different states of the reality of the premises, depending on the different needs for analysis time required by the different monitoring and follow-up procedures.

**Figure 7:**
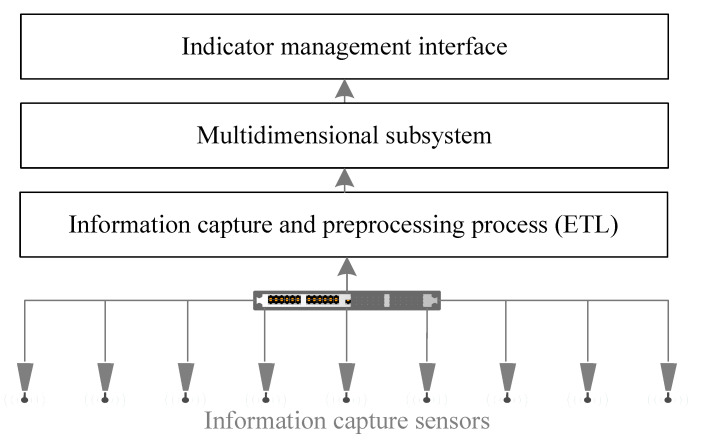
Outline of the online system for the monitoring and evaluation of environmental improvement plans (Extract, Transform and Load (ETL)).

Figure 7 shows the general structure of the system. The generation of information is based on the data capture made by the network of sensors in the hospital [68], which regularly collects the data, summarizing it and adapting it according to the needs of the multidimensional subsystem.

The information support of the multidimensional subsystem is the data obtained and processed from the sensor platform, which will finally be entered into a multidimensional database composed of two types of multidimensional variables: (i) additive variables that contain data summarized from the raw information, which contain aggregate information whose characteristic is that all its measures (contained indicators) correspond to additive functions such as those used in [81], (ii) classic multidimensional variables, which will be generated from queries made on the additive variables, but whose purpose is the computation of measures that cannot be expressed based on additive functions, which generates a significantly efficient and fast environment for the management online for this type of information.

The so-called indicator management interface corresponds to software components intended for managers and researchers, which will provide them with access to the information collected by incorporating graphical and analytical functionalities, in the approach known in the field of management as control panel [82].

### 5.3. Multidimensional Sub-System

As previously explained, the indicators of environmental well-being correspond mainly to numerical, continuous, or discrete variables, which are recorded with a frequency that varies according to the type of measurement instrument. They are mostly measurements that are obtained through sensors, in a real number format, on sequences of data measured at regular time intervals of one minute. All this information is recorded and managed jointly and integrated with information regarding the type of instrument used, the physical spaces, as well as the activities carried out in said spaces. As part of the online monitoring system, it receives requests from the indicator management interface.

Regarding the indicators, depending on their complexity and properties, it is possible to classify them into two types:

- Additive (or fractional) indicators: These are those in which the computation formula complies with the additivity property [83] or if it is possible to break it down into simpler expressions that do. This allows them to be obtained directly through a single OLAP operation from a multidimensional cube with aggregated information.
- Non-additive indicators: These are those indicators whose formula does not admit a decomposition into additive components. From this group, we will be particularly interested in those indicators whose computation is possible in two stages, where the first stage (much more demanding in terms of volume of data and number of computations) can be calculated from summaries contained in the database and, in the second stage, the partial results can be used to obtain the final value of the indicator.

**Figure 8:**
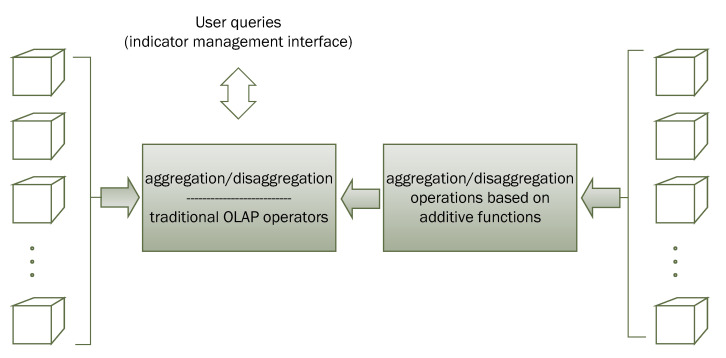
Multidimensional subsystem (On-Line Analytical Processing (OLAP)).

The above distinction is relevant because the response to a query will depend on the type of indicator involved. In effect, the queries on additive indicators will be made directly on an OLAP cube that contains a decomposition into additive parts of the indicator, obtaining a very significant impact on the efficiency of the queries due to the ability to use the additive properties of the different component parts (see Figure 8).

Queries on non-additive indicators will have different degrees of complexity. In the most desirable cases, its value can be obtained from fractional indicators that are contained in the database. For example, the case of $e^{\bar{x}}$ clearly depends on the average, which is an exponential indicator component, and yet it is a fractionable component.

**Figure 9:**
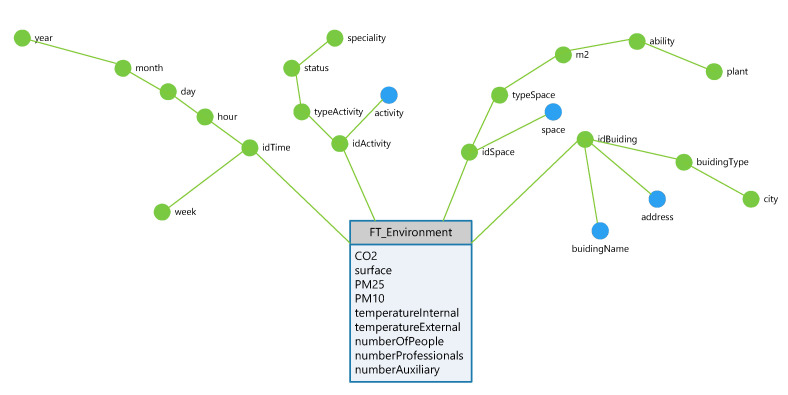
Environment multidimensional variable.

The system contemplates the management of the indicators contained in Table 2, which are, from the point of view of the data, totally and functionally dependent on the dimensions Time, Precinct, Activity, and Space. The attribute hierarchies of each dimension are as follows:

The multidimensional database will be composed of different variables, the most relevant for this work being the variable environment that houses the main indicators of Table 2. The conceptual model of the variable, expressed according to the representation proposed for Golfarelli and Rizzi [87] corresponds to Figure 9 while the Relational Online Analytical Processing (ROLAP) model is implemented on a relational database. Figure 9 and Table 3 clearly show the dimensions and their corresponding attribute hierarchies. Together, they constitute the dimensional space that, by selecting or transforming the attributes of the hierarchies, will allow the implementation of different data classification criteria.

The variables of the hierarchy of the time dimension correspond to the identification code (idTime), the hour, the day, the week, the month, and the year of a certain date; the variables of the hierarchy of the activity dimension correspond to its identification code (idActivity), its description (activity), its type, status, and specialty; the variables of the hierarchy of the space dimension correspond to its identification code (idSpace), its description (space), as well as its type, surface in m${}_{2}$, capacity, and floor plan; finally, the variables of the hierarchy of the enclosure dimension correspond to the identification code of the building (idBuilding), its name and address, as well as its type and its city.

On the dimension Space, whose schema is $E_{Space}=\left\{\mathrm{idSpace},m^{2},\ldots ,\mathrm{Plant}\right\}$, there is an order relation defined by the set:

$$\left\{\left(\mathrm{idSpace},m^{2}\right),\left(m^{2},\mathrm{Type}\right),\left(\mathrm{Type},\mathrm{Ability}\right),\left(\mathrm{Ability},\mathrm{Plant}\right)\right\}$$

Thus, the dimensional space of the space dimension (of degree five) will be the Cartesian product:

(1) $$ED_{space}=\prod_{i=1}^{5}\mathrm{Dom}\left(E_{j}\right),\;\;\;\;\;\;\;\;\;\;\;\;\;\;\;E_{j}\in E_{space}$$

Additionally, considering the dimensions Activity, Space, Building, and Time, made up of the attribute hierarchies of Table 3, we will define the multidimensional space of the variable Environment as the following Cartesian product:

(2) $$ED=\prod_{j\in D}^{}\prod_{i=1}^{g(ED_{j})}\mathrm{Dom}\left(C_{ij}\right)$$

where $g(ED_{j})$ corresponds to the degree of the *j*-th dimension, $\mathrm{Dom}(C_{ij})$ is the domain of the *j*-th attribute of the *i*-th dimension and *D* is the set of all dimensions:

$$D=\left\{Time,Building,Activity,Space\right\}$$

For its part, the scalar space corresponding to the multidimensional variable Environment will be made up of the following Cartesian product:

(3) $$SS=\prod_{i\in I}^{}\mathrm{Dom}\left(i\right)$$

where *I* is the set of all the indicators or measures considered in the multidimensional variable (Table 2).

The multidimensional variable Environment will be a function between the dimensional space and the scalar space (h:ED∼EE), in this way, the multidimensional variable environment, which we will also denote by *H*, will be a subset of the Cartesian product in ED and EE, on which it will be possible to carry out analytical activities using OLAP operators and ad hoc statistical tools.

Since two types of indicators are contemplated in this design, their computation will also require the use of two different types of Multiple Aggregation Function (MAF). As explained above, in the case of computation of additive or fractionable indicators, it will be possible to make a direct computation using an aggregation function that computes the necessary indicators, using the additive components of the indicators stored in the multidimensional variable. We will represent this aggregation function as follows:

(4) $$F\left(H\right)=(F_{1}\left(H\right),F_{2}\left(H\right),\cdots ,F_{n}\left(H\right))$$

For the computation of non-additive indicators, in those cases in which their computation can be partially supported by previous computations based on additive MAF stored in some multidimensional variable, a tabulation function will be established based on the results of a query on that variable, as follows:

(5) $$F^{\circ }N\left(H\right)=N\left(F\left(H\right)\right)=\left(N_{1}\left(F\left(H\right)\right),N_{2}\left(F\left(H\right)\right),\cdots ,N_{n}\left(F\left(H\right)\right)\right)$$

where *N* is an MAF that is not necessarily additive.

Although this query will require double counting, it will take advantage of the efficiency of the additive aggregation function used in the construction of the multidimensional variable as much as possible.

## 6. Discussion of Results

Considering the results of previous research and the reflections and learning achieved in them, it is possible to highlight results in different aspects, which are addressed in the following sections.

### 6.1. Feasibility of Technological Infrastructure

The design and implementation of specialized platforms made up of sensors, data networks, and computer services for the development of a cyber–physical system is in increasingly widespread use. Currently, it is a requirement for the competitiveNESs of an organization to incorporate new approaches and developments in the field of IoT, since with these technological tools it is possible to improve energy management.

From the point of view of economic and human resources, the installation and operation of the experimental monitoring platform proposed in this work is feasible. The Hospital Clínico Regional de Concepción Dr. Guillermo Grant Benavente is required to invest USD 70,500, which corresponds to 0.039% of the annual budget for the year 2021 [88]. This amount is low and is affordable by the Chilean public hospital.

For the levels of IAQ, the indicators (4–9) are shown in Table 2. Ind${}_{4}$ is recommend by UNE – EN 16798-3:2018 [36]. In the same way, Ind${}_{6}$ to Ind${}_{9}$ is recommended by Table I-1 std ASHRAE 62.1 2013 [85]. Finally, thermal comfort, temperature, and RH are recommended by Std ASHRAE 170-2013 [86], and clothes and physical activity are indicated as a function of node location and the outdoor temperature.

### 6.2. Installation of the Environmental Data Collection Platform

For the installation and start-up of a sensor node in the hospital, the following requirements must be met: (i) that there is prior availability or installation of network points with Power over Ethernet (PoE) to supply electricity and data network, (ii) that the physical installation of the sensor node is carried out according to the recommendations of the hospital staff, and (iii) that the initial logical configuration of the sensor node is based on the configuration of the Internet Protocol (IP) address of the server within each sensor node.

In relation to the research that was carried out in the hospital, the platform was installed in each hospital, the IP was assigned to the server and the physical installation was made in the server room. Then, we proceed with the configuration and start-up of the server, which consists of: (i) setting up the server on the hospital’s intranet, (ii) connectivity of the time synchronization service so that the platform has time synchronization time in the measurements executed, (iii) the configuration of access to the server from a remote location, (iv) the remote desktop access of the platform server, and (v) the network configuration of the sensor nodes in the platform.

Finally, it is possible to say that the installation of the platform prototype at the Hospital Clínico Regional de Concepción Dr.Guillermo Grant Benavente is suitable for operating in a real environment with measurements validated by external standard instruments [68]. As a result of the tests that were carried out, it can be inferred that the level of technological maturity of the platform is high since it is an innovative system that has been validated in an operating environment.

### 6.3. Computer Platform for Monitoring Environmental Indicators

The system proposed in this work is based on the management of data aggregations with additive properties, it allows to monitor the required indicators and considerably reduce the storage needs and the processing of the data generated by the sensor platform. This decrease is closely related to the attribute hierarchies used in the multidimensional variables of the multidimensional subsystem.

As an example, in Table 4, three variations in the specificity of the hierarchy of attributes of the time dimension of the data represented are considered. It is possible to appreciate the number of temporary registers necessary for the construction of the multidimensional variable. That is, when working with a specificity level at the minute level there is practically no aggregation.

In the case of this research, it is considered that the experimental platform consists of 15 nodes, its data collection capacity in a monthly period is 30 days and it has a periodicity of one minute, which allows obtaining a total of 648,000 data records monthly, which annually is equivalent to a total of 7,776,000 records. It is worth mentioning that if the number of sensors and monitored premises is increased, this annual figure will increase considerably.

A level of specificity is proposed by time slots and in special situations at the hour level, in Table 4 with which a huge impact is achieved on storage needs and data processing.

In the specific case of the ambient multidimensional variable shown in Figure 9, the variable allows monitoring of the behavior of the indicators according to their time slots. In addition, it takes advantage of the possibilities of decomposing them into a more specific set of additive functions, so it is possible to reduce the number of records needed according to the following expression:

(6) $$N^{\circ }Reg=nt\cdot nd\cdot nres\cdot SSA$$

where nt is the number of time slots, nd is the number of days considered, nres is the number of venues, and SSA is the sum of each of the types of activities carried out in each space of each enclosure.

For the experimental study, a data set corresponding to the measurements of the month of August 2021 was used as a sample. Because the operating time of the sensors was not continuous, the actual amount of recorded data reached 407,688 records. In the case under study, work was carried out in a single facility (nres) that has eight different hospital spaces, with six time slots (nt) in a period of 30 days (nd), and they perform an average of two activities per space. The total number of real records involved in the calculation of the indicators is approximately 6 · 31 · 1 · 12, that is, 2232 records.

In the case under study, this figure represents a 99.7% reduction in the number of records needed, which means that the monitoring and follow-up process drops to a fully manageable scale on a wide range of computer platforms, no matter how small and whatever its characteristics. If we add the use of a query language specially oriented to work on this type of platform [83], in addition to achieving much greater simplicity in formulating queries, direct access to the aggregated data will allow people without sophisticated knowledge to make database queries.

### 6.4. Limitations

The limitation of the study is that it only has the data generated in the sensors and actuators that were installed in different places of the hospital (see Table 1). A large amount of data related to temperature, relative humidity, pollutants, and particulate matter is obtained.

## 7. Conclusions

The introduction of using standards, such as the 2050 Energy Policy, the promulgation of laws and regulations related to the quality of public health services in Chile as well as the requirement of an adequately qualified administrative body related to the management of energy resources, allow the progressive implementation of methods and technology aimed at the efficient use of energy resources in hospitals, in a sustainable context. Following a sustainable context, the main limitation of this monitoring system is the implementation in a real environment of low-cost sensors with a certain accuracy for the measurement of the variables that determine the thermal comfort and air quality in a hospital.

As shown in Figure 4, in the present work a conceptual and cyber–physical system was proposed in which, based on the data collected by the sensors and actuators located in the care process of a hospital in the Bio-Bio, it is possible to measure with KPIs and monitor in real time the thermal comfort and the quality of the air inside the hospital premises.

Theoretical modeling of the main sustainable energy KPIs is created (see Table 2), related to critical aspects of patient care, such as energy consumption, installed power, level of thermal comfort, ambient temperature, and the number of infections. The results regarding the experimental construction of the multidimensional variables of the database coincide with expectations and open up a wide range of possibilities in the future in the development of new platforms for control, study, and monitoring that are more flexible and effective.

In addition, it is estimated that the cost of installing and putting into operation the experimental monitoring platform is feasible for a public health institution in Chile since it requires an investment of USD 70,500; which is equivalent to 0.039% of the annual budget of the Dr. Guillermo Grant Benavente Regional Clinical Hospital of Concepción. On the other hand, the implementation of this type of technology has other advantages such as energy savings that are generated by the reduction in the consumption of HVAC systems if the sensors of the monitoring network are installed in strategic locations, and that it helps to decision making at the managerial level.

This research can be extended to an online system with twelve different hospital facilities, it is estimated that it could have an average of 40 care spaces in each of them and two activities per facility, with which approximately 2,102,400 could be stored annually, versus the 504,576,000 that would require treatment without the use of additive indicators. This reduction would imply processing a smaller amount of data to train estimation structures that model the energy behavior of hospital facilities and that the related computer systems are not intensive in calculations, which would reduce the costs of operations. Future work also consists of evaluating the influence of thermal comfort and IAQ variables on energy consumption monitored automatically by smart field sensors of electricity or fuels, and including these new variables in the monitoring system. Furthermore, additional future work is the construction of automatic reports that allow visualizing hidden patterns in the stored data to detect abnormalities in energy consumption.

By incorporating new hospital facilities into the monitoring system, a network would be formed, where the multidimensional database could be managed in an independent data center. With this implementation, new knowledge related to more efficient energy management that improves the quality of care in public hospitals would be generated. It should be noted that in the event that data transmission takes place outside the hospital network, an information cybersecurity analysis would be required, such that unauthorized access to the hospital network or interruptions of information technology services are prevented.

## Figures and Tables

**Table 1:** The node sensors’ locations.

Node	Location	Floor	Category	Location Description
1	Data center	3	other	Data center
2	Ambulatory care center waiting room	4	waiting room	Neurology waiting room
3	Ambulatory care center waiting room	4	waiting room	Otolaryngology-bronchopulmonary waiting room
4	Ambulatory care center waiting room	4	waiting room	Ophthalmology waiting room
5	Ambulatory care center waiting room	2	waiting room	Cardiology waiting room
6	Ambulatory care center waiting room	2	waiting room	Cardiology waiting room
7	Auditorium—ambulatory care center	1	auditorium	Auditorium—ambulatory care center
8	Meeting room—ambulatory care center	6	other	Meeting room—ambulatory care center
9	Adult emergency waiting room—critical patient tower	1	waiting room	Adult medicine emergency-nursing station
10	Adult emergency waiting room—critical patient tower	1	waiting room	ER corridor
11	Adult emergency waiting room—critical patient tower	1	waiting room	Emergency waiting room
12	Adult emergency waiting room—critical patient tower	1	waiting room	Emergency waiting room
13	Emergency waiting room for children critical patient tower baseboard	−1	waiting room	Emergency waiting room for children critical patient tower baseboard
14	Emergency waiting room for children critical patient tower baseboard	−1	waiting room	Emergency waiting room for children critical patient tower baseboard
15	Sterilization tower critical patient baseboard	−1	services	Sterilization tower critical patient baseboard
16	Sterilization tower critical patient baseboard	−1	services	Sterilization tower critical patient baseboard
17	General pharmacy	1	services	Pharmacy warehouse-central: stores insulin and hormoNES that need refrigeration
18	General pharmacy	1	services	General pharmacy
19	Laundry	1	services	Laundry
20	Laundry	1	services	Laundry
21	Feeding	1	services	Food Center
22	Monoblock	2	waiting room	Pensioners—nursing station
23	Monoblock	2	hallway	pensioners-outside room 22
24	Monoblock	3	hallway	Aisle—medicine women
25	Monoblock	3	hallway	Aisle—ICU covid—room 39
26	Monoblock	2	hallway	Aisle (swap 1)
27	Monoblock	4	hallway	Aisle—delivery room
28	Monoblock	4	hallway	Aisle obstetric recovery
29	Monoblock	5	services	Milk dietary Services room
30	Monoblock	5	hallway	Pediatrics
31	Monoblock	5	hallway	Neonatology
32	Monoblock	4	hallway	Gynecology
33	Monoblock	3	hallway	Pediatrics—critical patient unit
34	Pharmacy mix	5	services	Pharmacy mix
35	Monoblock baseboard mechanical workshop	1	services	Monoblock workshop

**Table 2:** System indicators.

Indicador	Description	Additivity	Source
Ind${}_{1}$	Energy consumption per m${}^{2}$	Yes	[63]
Ind${}_{2}$	Energy consumption per user	Yes	[63]
Ind${}_{3}$	Installed power	Yes	[63]
Ind${}_{4}$	CO${}_{2}$	Yes	[67]
Ind${}_{5}$	Estimated CO${}_{2}$ generation per person	No	[84]
Ind${}_{6}$	Thermal comfort level	No	[31]
Ind${}_{7}$	Compliance level ASHRAE Standard 62.1 2013 and 170. 2017	No	[85,86]
Ind${}_{8}$	pm25 standard compliance level	No	[85]
Ind${}_{9}$	pm10 standard compliance level	No	[85]
Ind${}_{10}$	Indoor ambient temperature	Yes	[86]
Ind${}_{11}$	Outdoor ambient temperature	Yes	[86]
Ind${}_{12}$	Perception of thermal comfort vs. number of pathologies	No	Hospital staff
Ind${}_{13}$	Number of COVID-19 infections	Yes	Hospital staff
Ind${}_{14}$	Number of infections Influenza	Yes	Hospital staff
Ind${}_{15}$	Relationship between CO${}_{2}$ and viral transmission COVID	Yes	Hospital staff
Ind${}_{16}$	Relationship between CO${}_{2}$ and influenza viral transmission	Yes	Hospital staff

**Table 3:** Dimensions and hierarchies.

Dimension	Degree	Hierarchies
Time	5	$J_{\mathrm{Time}}:\mathrm{idTime}\to \mathrm{hour}\to \mathrm{day}\to \mathrm{month}\to \mathrm{year};\mathrm{Week}$
Building	2	$J_{\mathrm{Building}}:\mathrm{idBuilding}\to \mathrm{buildingType}\to \mathrm{city}$
Activity	3	$J_{\mathrm{Activity}}:\mathrm{idActivity}\to \mathrm{typeActivity}\to \mathrm{status}\to \mathrm{specialty}$
Space	5	$J_{\mathrm{Space}}:\mathrm{idSpace}\to \mathrm{typeSpace}\to m^{2}\to \mathrm{ability}\to \mathrm{plant}$

**Table 4:** Temporal granularity alternatives.

Specificity	Degree	Hierarchy	Cardinality
minute	6	minute→hour→tract→day→month→year	7,778,000
hour	5	hour→tract→day→month→year	133,920
tract	4	tract→day→month→year	32,400

## Data Availability

Not applicable.

## Abbreviations

The following abbreviations are used in this manuscript:

C${}_{2}$H${}_{5}$OH	Ethanol
CO	Carbon Monoxide
CO${}_{2}$	Carbon Dioxide
CPS	Cyber–Physical System
ETL	Extract, Transform and Load
GDP	Gross Domestic Production
GHG	Greenhouse Gas
HVAC	Heating, Ventilation, and Air Conditioning
IAQ	Indoor Air Quality
ICS	Information and Communications Systems
IoT	Internet of Things
IP	Internet Protocol
KPI	Key Performance Indicator
MAF	Multiple Aggregation Function
NES	National Electricity System
NO${}_{2}$	Nitrogen Dioxide
NOx	Nitrogen Oxides
OECD	Organisation for Economic Co-operation and Development
OLAP	On-Line Analytical Processing
OPC UA	Open Platform Communications Unified Architecture
PM${}_{10}$	Particulate Matter 10 micrometers or less in diameter
PM${}_{2.5}$	Particulate Matter 2.5 micrometers or less in diameter
PMV	Predicted Mean Vote
PoE	Power over Ethernet
PPD	Percentage of People Dissatisfied
RH	Relative Humidity
ROLAP	Relational Online Analytical Processing
SBC	Sustainable Building Certification
SDGs	Sustainable Development Goals
SO${}_{2}$	Sulfur Dioxide
UI	User Interface
VOC	Volatile Organic Compound
